# Supplementary material for: Whole-exome sequencing of the mummified remains of Cangrande della Scala (1291–1329 CE) indicates the first known case of late-onset Pompe disease
Source: Sci Rep. 2021 Oct 26;11:21070. doi: 10.1038/s41598-021-00559-1 (PMC8548527; doi:10.1038/s41598-021-00559-1)

Whole-exome sequencing of the mummified remains of Cangrande della Scala (1291–1329 CE) indicates the first known case of late-onset Pompe disease

Barbara Iadarola[1†], Denise Lavezzari[1†], Alessandra Modi[2], Chiara Degli Esposti[1], Cristina Beltrami[1], Marzia Rossato[1], Valentina Zaro[2], Ettore Napione[3], Leonardo Latella[4], Martina Lari[2], David Caramelli[2]*, Alessandro Salviati[1], Massimo Delledonne[1]*

[1]Department of Biotechnology, University of Verona, Strada Le Grazie 15, 37134, Verona, Italy; barbara.iadarola@univr.it; denise.lavezzari@univr.it; chiara.degliesposti@univr.it; cristina.beltrami_01@univr.it; marzia.rossato@univr.it; alessandro.salviati@univr.it; massimo.delledonne@univr.it

[2]Department of Biology, University of Florence, Via del Proconsolo 12, 50122, Florence, Italy; alessandra.modi@unifi.it; valentina.zaro@unifi.it; martina.lari@unifi.it; david.caramelli@unifi.it

[3]UNESCO office, Municipality of Verona, Piazza Bra 1, 37121, Verona, Italy; ettore.napione@comune.verona.it

[4]Department of Zoology, Natural History Museum of Verona, Lungadige Porta Vittoria 9, 37129, Verona, Italy; leonardo.latella@comune.verona.it

† B.I. and D.L contributed equally.

* Correspondence: massimo.delledonne@univr.it, Phone: +39 045 8027962; david.caramelli@unifi.it, Phone: +39 055 2757744.

Supplementary Figure S1. Properties of the library prepared from bone DNA without uracil-DNA glycosylase treatment.

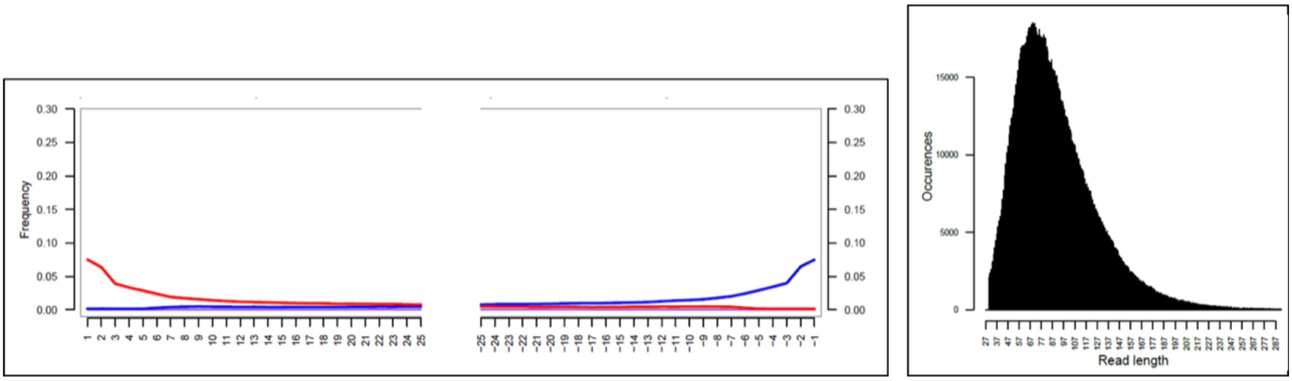

Supplementary Figure S2. Properties of the library prepared from bone DNA with partial uracil-DNA glycosylase treatment.

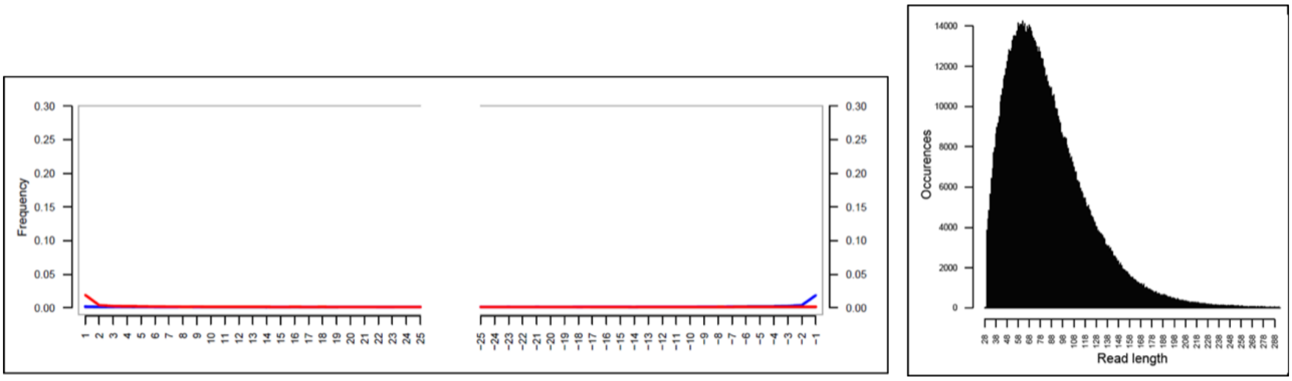

Supplementary Figure S3. Properties of the exome library.

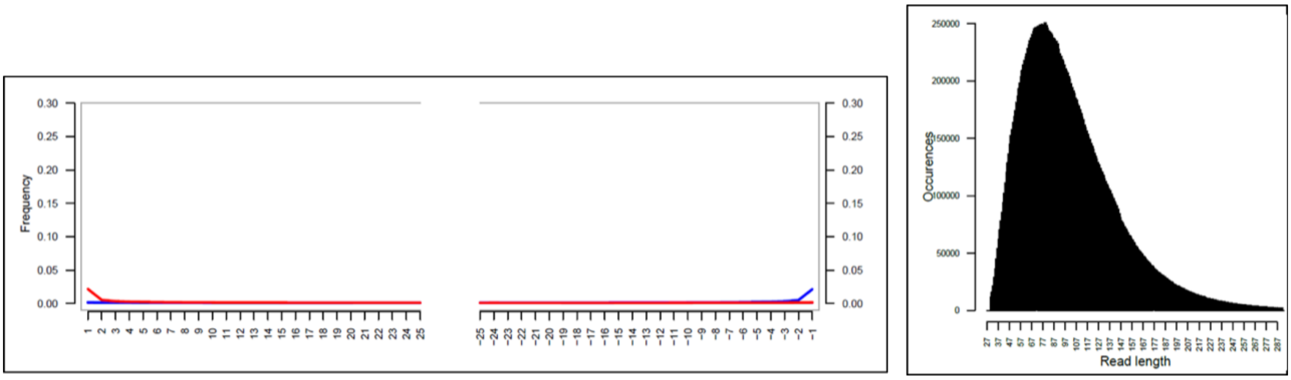

Supplement: Supplementary file 1 — Supplementary Figures. [file 41598_2021_559_MOESM1_ESM.pdf]
